# Healthy, mtDNA-mutation free mesoangioblasts from mtDNA patients qualify for autologous therapy

**DOI:** 10.1186/s13287-019-1510-8

**Published:** 2019-12-21

**Authors:** Florence van Tienen, Ruby Zelissen, Erika Timmer, Marike van Gisbergen, Patrick Lindsey, Mattia Quattrocelli, Maurilio Sampaolesi, Elvira Mulder-den Hartog, Irenaeus de Coo, Hubert Smeets

**Affiliations:** 1 Department of Clinical Genetics, Maastricht University Medical Centre+, Maastricht, The Netherlands; 2 School for Developmental Biology and Oncology (GROW), Maastricht University Medical Centre+, P.O. box 616, 6200MD Maastricht, The Netherlands; 3 School for Mental Health and Neurosciences (MHeNS), Maastricht University Medical Centre+, Maastricht, The Netherlands; 4 Department of Genetics and Cell Biology, Division Clinical Genomics, Maastricht University Medical Centre+, Maastricht, The Netherlands; 5 Department of Radiation Oncology (MaastRO Lab), Maastricht University Medical Centre+, Maastricht, The Netherlands; 6 Translational Cardiomyology, Department of Development and Regeneration, KU Leuven, Leuven, Belgium; 7 Center for Genetic Medicine, Northwestern University, Chicago, USA; 8 Human Anatomy Unit, Department of Public Health, Experimental and Forensic Medicine, University of Pavia, Pavia, Italy; 9 Department of Pediatric Surgery, Erasmus Medical Center, Rotterdam, The Netherlands; 10 Neuromuscular and Mitochondrial research center (NeMo), Rotterdam/Maastricht, The Netherlands

**Keywords:** mtDNA mutation, Mesoangioblasts, Muscle regeneration

## Abstract

**Background:**

Myopathy and exercise intolerance are prominent clinical features in carriers of a point-mutation or large-scale deletion in the mitochondrial DNA (mtDNA). In the majority of patients, the mtDNA mutation is heteroplasmic with varying mutation loads between tissues of an individual. Exercise-induced muscle regeneration has been shown to be beneficial in some mtDNA mutation carriers, but is often not feasible for this patient group. In this study, we performed in vitro analysis of mesoangioblasts from mtDNA mutation carriers to assess their potential to be used as source for autologous myogenic cell therapy.

**Methods:**

We assessed the heteroplasmy level of patient-derived mesoangioblasts, isolated from skeletal muscle of multiple carriers of different mtDNA point-mutations (*n* = 25). Mesoangioblast cultures with < 10% mtDNA mutation were further analyzed with respect to immunophenotype, proliferation capacity, in vitro myogenic differentiation potential, mitochondrial function, and mtDNA quantity.

**Results:**

This study demonstrated that mesoangioblasts in half of the patients contained no or a very low mutation load (< 10%), despite a much higher mutation load in their skeletal muscle. Moreover, none of the large-scale mtDNA deletion carriers displayed the deletion in mesoangioblasts, despite high percentages in skeletal muscle. The mesoangioblasts with no or a very low mutation load (< 10%) displayed normal mitochondrial function, proliferative capacity, and myogenic differentiation capacity.

**Conclusions:**

Our data demonstrates that in half of the mtDNA mutation carriers, their mesoangioblasts are (nearly) mutation free and can potentially be used as source for autologous cell therapy for generation of new muscle fibers without mtDNA mutation and normal mitochondrial function.

## Introduction

Mitochondria generate the bulk of cellular energy (ATP), and each mitochondrion contains multiple copies of mitochondrial DNA (mtDNA). Together with nuclear-encoded proteins, the mtDNA encodes the subunits of the ATP-generating oxidative phosphorylation (OXPHOS) system. Pathogenic heteroplasmic (mixture wild-type/mutant mtDNA) mtDNA mutations can cause OXPHOS dysfunction and severe multisystem disorders (frequency 1/5000) [1]. High-energy-requiring tissues like muscle and brain are most severely affected. Progressive myopathy and exercise intolerance occur in > 50% of mtDNA mutation carriers, and aging appears to play a major role as well, since the percentage of myopathy patients drastically increases with age [2, 3], making these the most debilitating symptoms for these patients [4].

One of the most common mtDNA mutations giving rise to myopathy, either isolated or as part of a clinical spectrum, is the m.3243A>G mutation in the dihydrouridine loop of mitochondrial tRNA Leu (UUR) [5]. But also other tRNA Leu point-mutations, as well as mutations in protein-encoding and in other tRNA genes, have been observed in patients with mitochondrial myopathy [2, 3]. These mutations are generally heteroplasmic, and large differences in mutation load between tissues were seen. Post-mitotic tissues, such as skeletal muscle, generally possess the highest mutation load. In addition to variability between tissues, the mutation load can also vary in time or between muscle fibers of a single skeletal muscle [3, 6]. If the mutation load exceeds a certain tissue-specific threshold, the mitochondrial oxidative phosphorylation (OXPHOS) system will become deficient, resulting in diminished oxidative capacity and pathology leading to clinical symptoms.

One of the key problems for mtDNA patients is the lack of effective therapy. No pharmaceutical drugs have been shown to treat mtDNA diseases effectively in clinical trials [7]. Alternatively, physical exercise has shown some success in inducing muscle regeneration and improving OXPHOS capacity, but this is seriously hampered by the exercise intolerance and exercise-induced lactate increase in mtDNA mutation carriers and patients. Both resistance and endurance exercise protocols have been applied in mtDNA patients [8, 9]. In contrast to endurance exercise that predominantly induces mitochondrial biogenesis, resistance type exercise increases muscle mass by inducing regeneration through proliferation and differentiation of myogenic precursors like satellite cells and other stem cells [10–12]. Satellite cells are regarded as the major myogenic precursors and in carriers of the sporadic m.12315G>A or m.12320A>G mutation, they have been shown to be nearly all homoplasmic wild-type despite a high mutation load of 94% in skeletal muscle. Moreover, induction of muscle regeneration following muscle damage demonstrated to trigger generation of nearly all homoplasmic wild-type COX-positive muscle fibers in these persons [13–15]. Large-scale mtDNA deletions are lost during satellite cell to myoblast transition in a number of CPEO patients with > 3 kb mtDNA deletions and mutation load in skeletal muscle varying between 10 and 67% [16]. However, the therapeutic potential of satellite cells is hampered by their poor ex vivo proliferation capacity and requirement for intra-muscular delivery [17].

In recent years, other types of stem cells with myogenic potential have been identified [18], one being pericytes, also called mesoangioblasts (MABs), which are mesoderm-derived (CD13^+^/CD44^+^/NG2^+^) stem cells that lie underneath the basal lamina of the small vessels of muscle fibers [19, 20]. Since there is no specific MAB marker, they are characterized based on a combination of markers. MABs have high myogenic potential (20–40%) and induce muscle regeneration either directly by fusion with damaged muscles or by contributing to the satellite cell pool [21]. Moreover, they can be efficiently expanded ex vivo; MABs express multiple growth factors and ß2 and α4 integrins, which are proteins used by leukocytes to adhere and cross the endothelium, allowing systemically intra-arterial administration [17, 22, 23]. Muscle regeneration by systemic injection of allogeneic MABs has been reported to induce muscle regeneration and partly compensation for the defect in mice and dog models of Duchenne muscular dystrophy (DMD) [22, 24]. The success of MAB transplantation in animal models with respect to safety, proliferative capacity, and myogenic potential has led to a phase I/II clinical trial with donor MABs for DMD [25], which showed that the method was feasible and relatively safe. The drawback of an allogeneic cell therapy is the requirement for immunosuppression, which would be circumvented by using autologous MABs. Because of the therapeutic potential of MABs in myogenic stem cell therapy and the identification of satellite cells and myoblast clones with low/absent mtDNA mutation load in mtDNA mutation carriers [14, 16, 26], we assessed if MABs from carriers of a maternally inherited mtDNA point-mutation or a sporadic mtDNA deletion could potentially be used for autologous cell therapy.

## Materials and methods

### Study design

Participants of this explorative study enrolled at the study site Erasmus MC (NL). All individuals underwent a vastus lateralis muscle biopsy under local anesthesia for other (diagnostic) analyses. Written informed consent was obtained from all participants, and this study was approved by the medical ethical committee of the Erasmus MC, The Netherlands. A total of 13 carriers of a heteroplasmic tRNA Leu mutation in the mtDNA were included: 9 m.3243A>G carriers, 2 persons (mother and daughter) with a m.3271 T>C mutation, and 2 persons (mother and daughter) with a m.3291 T>C mutation. For the heteroplasmic m.11778G>A mutation in the MT-ND4 gene, 2 siblings were included. For the heteroplasmic m.8363G>A mutation in tRNA Lys, 4 cousins were included. Clinical characteristics of the individuals are shown in Table 1. The point-mutations were all maternally inherited. Heteroplasmic large-scale deletions were observed in 6 chronic progressive external ophthalmoplegia (CPEO) patients. These were sporadic and POLG mutations were excluded. POLG sequence analysis and patient verification were performed according to clinical diagnostic standards (data not shown). The phenotypic characteristics of all participants are presented in Table 1.

**Table 1:** Clinical and molecular characteristics mtDNA mutation carriers

Patient ID	Age	Sex	mtDNA mutation	% sk. muscle	% MABS	Clinical features
M1^a^	37	M	tRNA Lys m.8363G>A	46	< 1	Mild EI, cephalia
M8^a^	40	F		51	6.0	EI, ataxia, dysarthria
M18^a^	35	F		87	78	Spinocerebellar ataxia with dysarthria
M20^a^	47	F		90	82	EI, axonal polyneuropathy, dysarthria
M5^b^	27	F	tRNA Leu m.3243A>G	40	9.4	EI, restless legs, cephalia (migraine like)
M6^b^	21	M		93	3.4	MELAS, bradyphrenia, mental deterioration
M9^c^	59	F		15	1.6	DM, hearing loss, cerebellar ataxia, slight mental deterioration
M10^c^	33	F		28	< 1	Discrete cerebellar ataxia
M132	61	F		62	9.1	Cerebellar ataxia, hemianopsia, CVA hemiparesis right, motor aphasia, DM, EI, seizures
M133	38	F		73	5.5	DM, hearing aid, CMP, EI, migraine-like headaches, proximal weakness, reactive depression
M137	58	M		80	22	Deafness, EI, CVA, PNP, DM, hypertension, Dupuytren
M19	43	F		79	64	MIDD, anorexia
M32	40	M		80	67	MIDD, nephropathy, myopathy, HCM
M2	22	F	tRNA Leu m.3271 T>C	100	96	Myopathy, EI
M22^e^	51	F		73	41	Headaches
M11^d^	11	F	tRNA Leu m.3291 T>C	94	73	EI, CMP, myopathy
M17^d^	34	F		62	1.6	EI, hearing loss, mental retardation
M34 ^f^	63	M	ND1 m.11778G>A	89	68	LHON
M37^f^	69	F		53	57	No complaints
M28	58	M	4977 bp deletion	ND	< 1	CPEO, myopathy, glaucoma, hearing loss, hypoventilation
M23	57	F	4977 bp deletion	41	< 1	CPEO/KSS, myopathy, swallowing problems, CMP, EI, PNP
M134	60	F	4977 bp deletion	60	< 1	CPEO, proximal discrete myopathy
M33	66	F	4977 bp deletion	45–50	< 1	CPEO, myopathy
M24	21	F	6 kb deletion	ND	< 1	CPEO, EI, dyspepsia, dysphagia
M07	42	M	3.5 kb deletion	60–70	< 1	CPEO/KSS, opticopathy, axonal motor polyneuropathy

*Age* age at muscle biopsy, *% sk. muscle* mean mtDNA mutation load in skeletal muscle, *% MABs* mean mtDNA mutation load in mesoangioblasts, *DM* diabetes mellitus, *EI* exercise intolerance, *MELAS* mitochondrial encephalopathy with lactic acidosis and stroke-like episodes, *MIDD* maternally inherited diabetes and deafness, *HCM* hypertrophic cardiomyopathy, *PNP* polyneuropathy, *CVA* cerebrovascular accident, *LHON* Leber’s Hereditary Optic Neuropathy, *CPEO* chronic progressive external ophthalmology, *KSS* Kearns-Sayre syndrome, *CMP* cardiomyopathy, *ND* not determined

^a^M8 and M20 are siblings and cousins of M1 and M18

^b^M5 and M6 are siblings

^c^M9 is mother of M10

^d^M17 is mother of M11

^e^M22 is mother of M02

^f^M34 and M37 are siblings

### Mesoangioblast isolation and culture

The muscle fragment was collected in mesoangioblast culture medium: IMDM medium containing 10%FBS (Bodinco), 0.1% gentamycin, 1X glutamine, 1X sodium pyruvate, 0.2% 2-mercapto ethanol, 1x Insulin-Transferrin-Selenium, 1X Non-Essential Amino Acids, and 5 ng/ml human FGF-2 (Miltenyi Biotec). All materials were obtained from Thermo Scientific, unless stated otherwise. Mesoangioblasts from a vastus lateralis skeletal muscle biopsy were isolated and cultured as described before [27]. In brief, skeletal muscle biopsies were rinsed with PBS, cut in small fragments, and plated on a type I collagen-coated dish with a few drops of the aforementioned medium. From the muscle biopsy, fibroblasts spread out while mesoangioblasts poorly adhere to these fibroblasts. After 10–14 days during which medium was regularly added, the medium containing mesoangioblasts was transferred to a new dish (5000/cm^2^) and MABs were cultured as attaching cells. Alternatively, outgrowth of the muscle biopsies was trypsinized after 10–14 days and seeded to a new dish at a 10,000 cells/cm^2^. The following 2 days, the medium containing mesoangioblasts was transferred to a new dish (5000/cm^2^) and MABs were further cultured as attaching cells.

### Mesoangioblast characterization and single-cell collection

Alkaline phosphatase-positive cells were collected via sorting using PE-labeled anti-alkaline phosphatase (R&D) as described [28] on a FACS ARIA (BD). Alternatively, cells were stained using the Alkaline phosphatase staining kit (Stemgent), and AP-positive MABs were collected manually using a micromanipulator as described previously [29]. For MAB characterization, FACS analysis of 10,000 cells was performed using PE-labeled CD13, CD44, CD45, CD34, CD31, and CD56 (Miltenyi Biotech). Depletion of CD56+ cells was performed using magnetic-activated cell sorting (MACS) using CD56 microbeads according to the manufacturers’ protocol (Miltenyi Biotech).

### DNA isolation

Genomic and mtDNA from cell pellets and tissues was isolated using the Wizard Genomic DNA isolation kit (Promega) according to the manufacturer’s protocol. Single cells were lysed by adding alkaline lysis buffer containing 50 mM dithiothreitol (DTT) (Pharmacia Biotech) and 200 mM NaOH (Sigma), followed by 15-min incubation at 65 °C.

### Genetic analyses of mtDNA mutations

After cell lysis of single cells, the mtDNA mutation load of the m.3243A>G, m.8363G>A, m.3271 T>C, and 3291 T>C mutation was analyzed by directly performing PCR I on the GeneAmp PCR System 9700 (Perkin-Elmer Applied Biosystems) in a total volume of 50 μl. The PCR mix contained Tricine (20 mM pH 4.95 (Sigma)) for neutralization of the alkaline lysis buffer, 1× PCR buffer, 1 U of Taq DNA polymerase, 0.06 μM forward primer, 0.3 μM reverse primer, MgCl_2_, and 0.1 mM dNTP (Pharmacia). First-round PCR started with 5-min denaturation at 94 °C followed by 34 cycles (or 38 cycles for single cells) of 1 min 92 °C, 45 s at Tm primer (see Additional file 1: Table S1) and 45 s at 72 °C, followed by 7 min at 72 °C. Fifteen microliters of first-round amplification product was used for one final amplification cycle containing labeled primer (Additional file 1: Table S1) using PCR temperatures and times/cycle as for PCR I. Fifteen microliters of the labeled second-round PCR product was digested in a total volume of 50 μl containing 10 U restriction enzyme (10 U/ml; Biolabs). After digestion, samples were purified using the innoPREP PCR pure kit (Analytik-Jena) and analyzed by capillary electrophoresis on an ABI Prism 3730 Genetic Analyzer followed by GeneScan Analysis 3.7 software package (Applied Biosystems). Specific details for the detection of each point-mutation (primer sequences, Tm, MgCl2 concentration, restriction digestion enzyme, expected sizes mutant and wild-type digested PCR products) are described in Additional file 1: Table S1. Real-time quantitative PCR on an ABI 7900HT machine using a wild-type-specific and m.11778G>A mutation-specific primer was used for quantification of the m.11778G>A mutation load. After single-cell lysis, 100 mM tricine (Sigma) and water were added to a total volume of 10 μl, of which 2.5 μl was used per PCR reaction. Real-time PCR amplification was performed in a 12.5 μl reaction containing 1x Sensimix Sybr Hi-Rox reagents (Bioline), 375 nM forward primer (mutant or wild-type), and 375 nM reverse using the following settings: 10-min denaturation at 95 °C, followed by 45 cycles of 15 s 95 °C and 1 min 67 °C. Semi-quantitative analysis of large-scale mtDNA deletions was assessed by long-range PCR amplification of the mtDNA. Per reaction, 1x GC-buffer, 1 U Phusion Hot Start DNA polymerase II, 0.1 mM dNTP (Pharmacia), and 0.5 uM forward primer. PCR started with 30-s denaturation at 98 °C, 32 cycles of 10 s 98 °C, and 8 min 15 s 72 °C, followed by 10 min at 72 °C. Subsequent PCR analysis was performed to verify the m.8482_m.13460 deletion of 4977 bp (nucleotide position based on NC_012920.1). PCR mix contained 1x Amplitaq 360 master mix, 0.6 μM forward primer, and 0.6 μM reverse primer. PCR started with 5-min denaturation at 95 °C, 40 cycles of 30 s 95 °C, 30 s 60 °C, and 1 min 72 °C, followed by 5 min at 72 °C.

### mtDNA copy number analysis

The mtDNA copy number was determined by comparing the ratio of mtDNA (D-loop) to nuclear DNA (B2M). Per reaction, 5 ng DNA was amplified in a 12.5 μl reaction containing 1x Sensimix Sybr Hi-Rox reagents (Bioline) and 375 nM of forward and reverse primer. Real-time quantitative amplification was performed on a ABI 7900HT using the following settings: 10 min 95 °C, 40 cycles of 15 s 95 °C and 1 min 60 °C.

### Proliferation capacity

Mesoangioblasts at passage 4 were seeded at a density of 5000/cm^2^ in mesoangioblast culture medium and passaged every 3–4 days. At each passage, cell number was assessed in duplicate using a Fuchs-Rosenthal hemocytometer. Population doubling level = 3.32 (log viable cells at harvest − log seeded cells).

### Myogenic differentiation

At confluence, myogenic differentiation of mesoangioblasts was induced by DMEM supplemented with 2% horse serum and 1% pen/strep for 10 days. After 5 min 3.7% formaldehyde (Sigma-Aldrich) fixation, PBS rinse, permeabilization in 0.2% Triton in PBS for 30 min and 1 h blocking in 1% BSA (Sigma), cultures were stained with MF20 antibody (DSHB) 1:10 in 0.05% triton/PBS overnight at 4 °C. Subsequently, the cells were rinsed three times with PBS, incubated with GAM-AF488 1:1000 for 1 h, rinsed three times with PBS, and mounted with DABCO and DAPI in glycerol (Sigma-Aldrich). In vitro myogenesis was calculated as the number of nuclei in MF20-positive fibers divided by total number of nuclei per field. A minimum of 100 nuclei were counted from ≥ 3 randomly chosen fields.

### OXPHOS capacity

Mesoangioblasts were seeded at 20,000 cells/well 1 day prior to analysis, and the OXPHOS capacity was assessed using the Seahorse XF Cell Mito Stress Test kit (Agilent) on the Seahorse XF96 extracellular flux analyzer (Agilent) according to the manufacturer’s protocol. For the mitochondrial stress test, the following injections were subsequently used: 1 μM oligomycin, 0.6 μM carbonyl cyanide-*4*-(trifluoromethoxy) phenylhydrazone (FCCP), and a combination of 1 μM rotenone and 1 μM antimycin A.

### Statistical analyses

A beta linear regression model was constructed to describe the myogenic fusion index (%). A hierarchical model was built, including interactions, allowing for sex, the age at muscle biopsy, and the mtDNA mutation load in mesoangioblasts (log scale) to be taken into account. The inference criterion used for comparing the models is their ability to predict the observed data, i.e., models are compared directly through their minimized minus log-likelihood. When the number of parameters in models differs, they are penalized by adding the number of estimated parameters, a form of the Akaike information criterion (AIC) [30]. All statistical analyses presented were performed using the freely available program R [31] and the publicly available libraries “gnlm” [32].

## Results

### Mutation load mtDNA point-mutations in skeletal muscle and mesoangioblasts

**Fig. 1:**
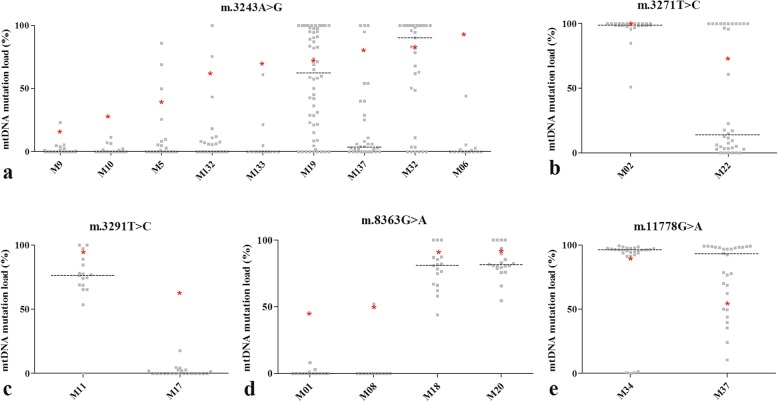
mtDNA mutation load per mtDNA point-mutation in single mesoangioblasts. The mtDNA mutation load in single mesoangioblasts in carriers from 5 different mtDNA point-mutations: **a** m.3243A>G, **b** m.3271 T>C, **c** m.3291 T>C, **d** m.8363G>A, and **e** m.11778G>A. Each gray dot represents the mtDNA mutation load in a single mesoangioblast (> 15 per person were analyzed). Asterisk indicates mean mtDNA mutation load in skeletal muscle. Dotted black line indicates median mtDNA mutation load analyzed in single mesoangioblasts

From all mtDNA point-mutation carriers included in this study, each familial mutation was detected in hair, blood, and/or urine. A *vastus lateralis* muscle biopsy was performed to establish the mtDNA mutation load in skeletal muscle and isolate MABs. From each subject, mesoangioblasts were stained with alkaline phosphatase (AP) and single AP-positive cells were collected using a micromanipulator. The mtDNA mutation load was analyzed in a minimal 15 single cells per subject. As shown in Fig. 1, for the majority of m.3243A>G mutation carriers, the mutation load was drastically lower in MABs compared with skeletal muscle of the same individual. For the m.8363A>G mutation, this was different. In two persons (M01, M08) with a skeletal muscle m.8363A>G load between 41 and 51%, the median MAB mutation load was 0%, while two other m.8363G>A carriers (M18 and M20) with a high mutation load in skeletal muscle had a comparable mutation load in MABs (respectively 89% and 91%). For the two other tRNA Leu mutations, m.3271 T>C and m.3291 T>C, mother and daughter were analyzed. In both cases, the daughters (M02 and M11) displayed a high mutation load in both skeletal muscle and MABs compared to their mothers. The m.11778G>A mutation was analyzed in MABs and skeletal muscle of two siblings, M34 and M37. M34 and M37 respectively carried > 90% and 53% of them.11778G>A mutation in muscle. The distribution of the m.11778G>A mutation load in single mesoangioblasts was different, as single M34 MABs were either > 90% or (near) wild-type, whereas single M37 MABs were more heterogeneous and displayed a spectrum between 0 and 100%.

### Large-scale deletions in skeletal muscle and mesoangioblasts

**Fig. 2:**
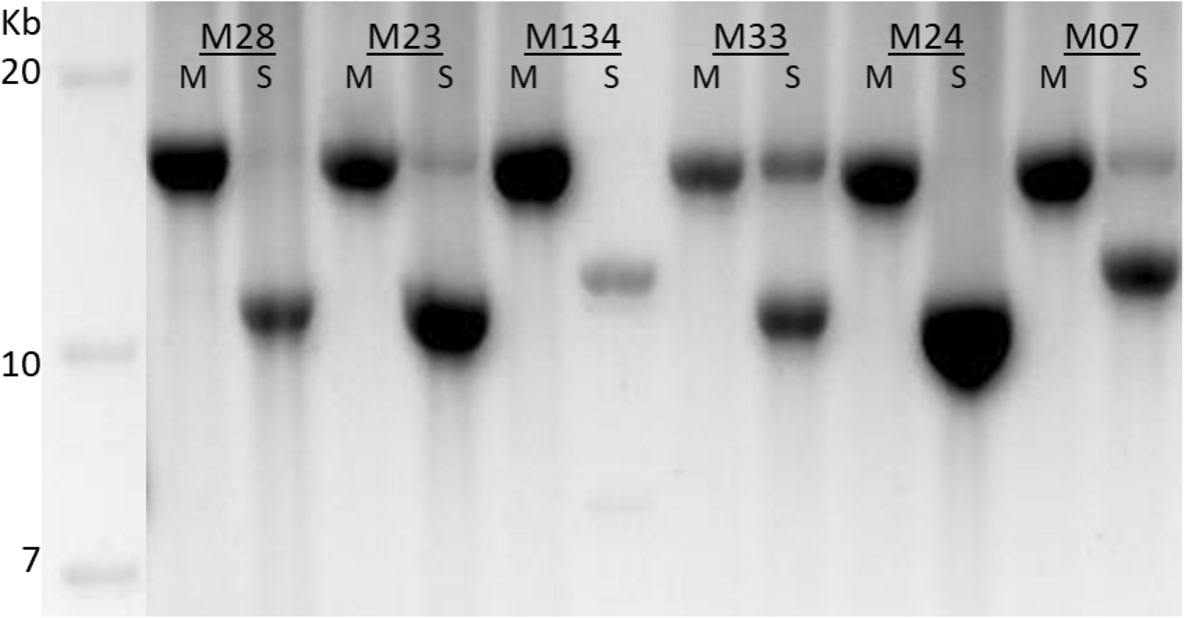
Semi-quantitative analysis of large-scale mtDNA deletions in mesoangioblasts and skeletal muscle. The 16.5 kb mtDNA was PCR amplified and analyzed on a 0.7% agarosegel. M, mesoangioblasts; S, skeletal muscle

For six carriers of a sporadic large-scale deletion in skeletal muscle, this deletion was undetectable following PCR amplification of the complete mtDNA in mesoangioblasts (see Fig. 2). Four of the six patients carried the most frequently occurring mtDNA deletion of 4977 bp. The m.8482_13460 breaking points were confirmed in the muscle tissue of these patients, but were also not detected in the isolated mesoangioblasts (data not shown). For patients M23 and M33, the large-scale deletion analysis was also performed in blood, but no large-scale deletions were detected as well (data not shown).

### Mesoangioblast characterization, proliferation, and myogenic differentiation capacity

**Fig. 3:**
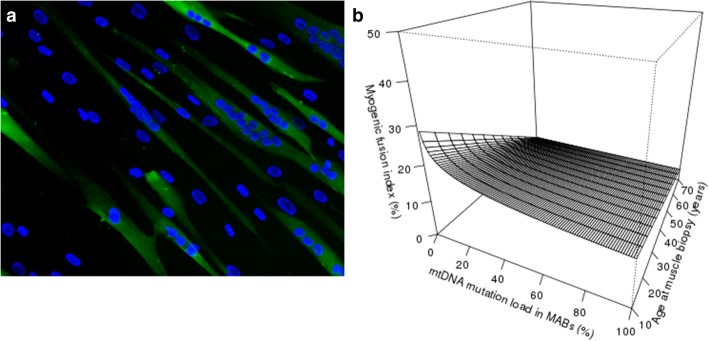
Myogenic potency of mesoangioblasts from mtDNA carriers. The myogenic potential was quantified following 10 days of differentiation in 2% horse serum containing medium and quantification of the myogenic fusion index, namely the number of nuclei (DAPI) in myosin-positive (MF20) muscle fibers per total number of nuclei per field. **a** Example image of 10 days differentiated MABs following MF20 immunostaining of myotubes (green) and DAPI nuclear stain (blue). **b** Interaction between age at biopsy and the mesoangioblast mtDNA mutation load (irrespective of type of mtDNA mutation) on the spontaneous myogenic potential of mesoangioblasts, using the best linear regression model obtained and described by *E*(Myogenic fusion index) = 0.146 − 0.00155 × age − 0.0394 × ln(MABs) + 0.00056 × age × ln(MABs)

The mesoangioblasts from mtDNA mutation carriers (M06, M10, M19, M34) proliferated similar to controls (19 ± 4 population doublings, starting from passage 4). Mesoangioblasts isolated from subjects between 21 and 63 years old were analyzed, and no correlation between age at biopsy and proliferation capacity was observed. In mesoangioblasts with < 10% mtDNA mutation load, repetitive analysis of the mutation load at passages 4, 6, 9, and 15 showed a maximal difference in mutation load of 2.4%. Immunophenotype analysis was performed, and the MAB cultures were positive for CD13 (99.6 ± 0.5%) and CD44 (99.4 ± 0.8%), and negative (< 5%) for CD31 (0.6 ± 0.5%), CD34 (1.0 ± 1.9%), and CD45 (0.5 ± 0.5%) (*n* = 13) (see Additional file 1: Table S3). When analyzed at passage 2, two cultures displayed > 5% CD34 expression, which was lost at passages 5 and 8, respectively. In addition, analysis of CD56 showed > 5% CD56+ cells in 6 out of 13 cultures. Magnetic activated cell sorting (MACS) using CD56+ beads demonstrated efficient depletion of CD56^+^ cells (< 5%). To assess the myogenic potency of MABs isolated from mtDNA mutation carriers, MABs from patients of varying age and with varying mtDNA mutation load were in vitro differentiated in multinucleated myotubes after culture in myogenic medium. The observed percentage of multinucleated fibers ranged between 0 and 37.1% (*n* = 18) (Additional file 1: Table S4). The best linear regression model included an interaction between age at biopsy and the log mtDNA mutation load in mesoangioblasts, as shown in Fig. 3. Analysis of the mtDNA mutation load after in vitro myogenic differentiation was not significantly changed 1.5% ± 1.7% in MAB-derived myotubes compared with undifferentiated MABs (*n* = 4).

### Mitochondrial OXPHOS capacity and mtDNA copy number in mesoangioblasts

**Fig. 4:**
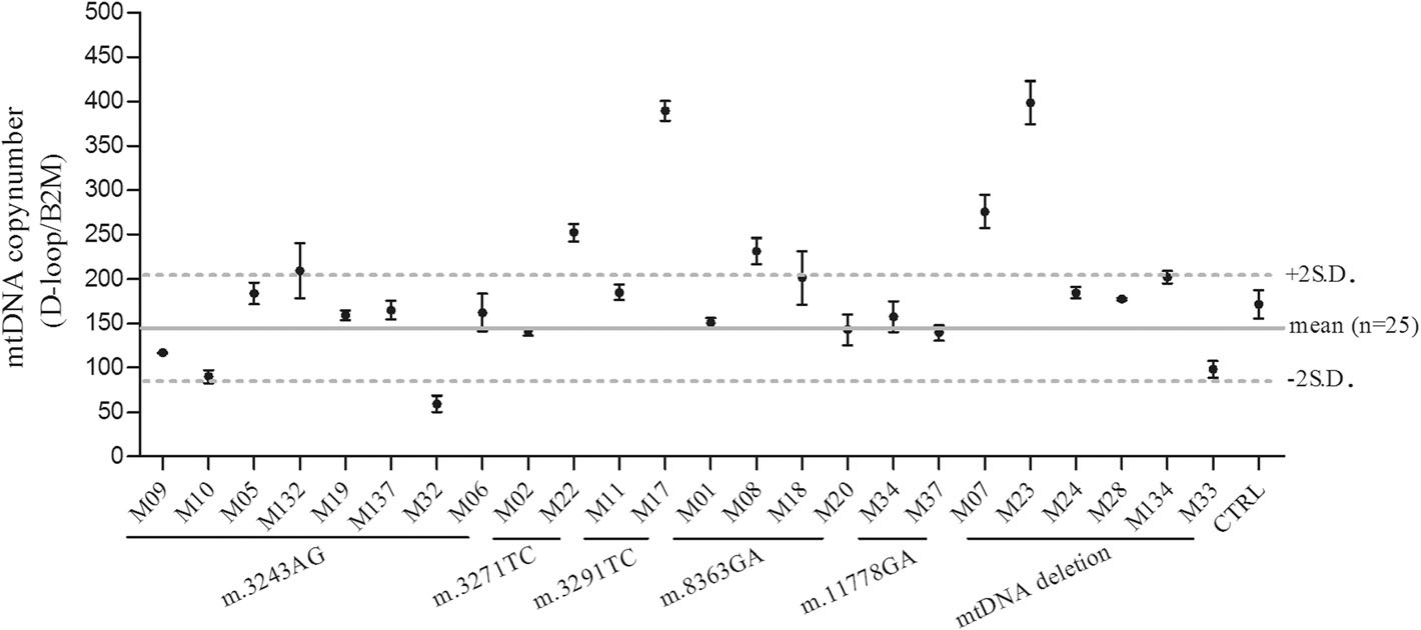
Mesoangioblast mtDNA copy number. The mean mtDNA copy number per cell line was determined by qPCR analysis of mtDNA D-loop and nuclear B2M. Per sample, the mean mtDNA copy number ± S.E.M. is shown. The solid line represents the mean mtDNA copy number calculated from all samples (*n* = 27), and dashed lines indicate mean ± 2 S.D.

**Fig. 5:**
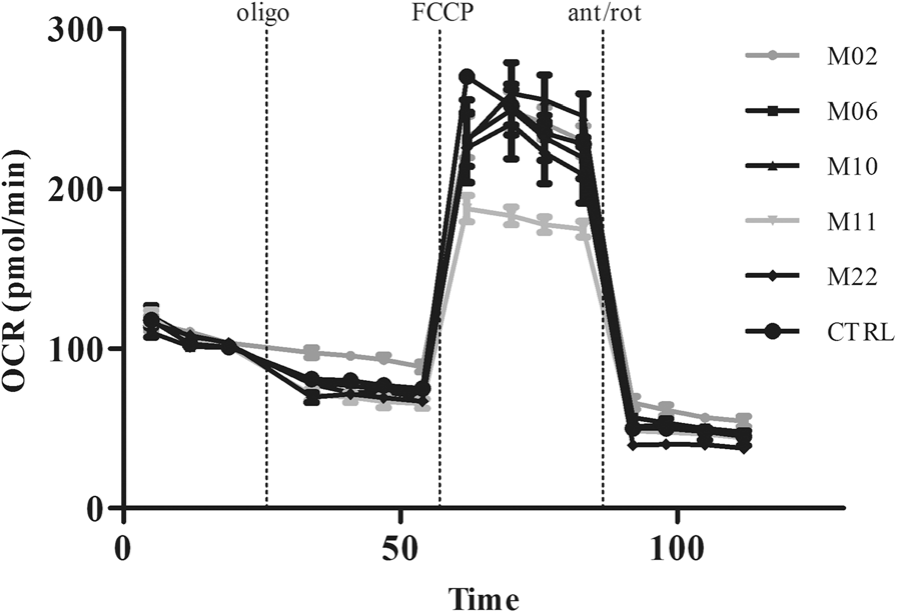
Mitochondrial function. Mitochondrial respiration in MABs of tRNAleu mutation carriers was determined by measuring the oxygen consumption rate (OCR) in a Seahorse XF96 analyzer during treatment with oligomycin (oligo), FCCP and Antimycin/Rotenone (ant/rot), and corrected for cell number. Per cell line, 8 replicates were included; mean OCR ± S.D. is shown in figure

The mtDNA copy number was determined in all mtDNA mutation carriers and controls. As shown in Fig. 4, mesoangioblasts contain on average 148 ± 56 (mean ± S.D.) mtDNA copies per cell. No clear correlation between mesoangioblasts with a high or low mutation load could be observed, nor a correlation between type of mutation and mtDNA copy number. Mutations in tRNAleu are known to affect the oxygen consumption rate. We assessed the oxygen consumption rate in six mesoangioblast cultures of tRNAleu mutation carriers. Four had no or low mtDNA mutation load (< 10%) (M06, M10, M22, CTRL) and displayed normal mitochondrial function (Fig. 5), while two mesoangioblast cultures with a high > 80% mtDNA mutation load (M02 and M11) displayed aberrant mitochondrial function.

## Discussion

In 11 out of 19 mtDNA point-mutation carriers, the mean mutation load was more than 50% lower in mesoangioblasts compared with skeletal muscle. Moreover, the mesoangioblast mutation load was below 10% in 9 individuals (Fig. 1). This drastic difference in mutation load between mesoangioblasts and skeletal muscle was observed for all five mutations analyzed, but most frequently in carriers of the m.3243A>G mutation (7 of 9 cases). Moreover, large-scale deletion(s) were not observed in mesoangioblasts of any of the mtDNA deletion carriers. At this moment, we cannot exclude that the mtDNA deletion was present in MABs when residing quiescent in the skeletal muscle. The mtDNA mutation may also be lost during the first proliferation step ex vivo, as has been reported by Spendiff et al. for satellite cells from carriers of a large-scale mtDNA deletion. In most of the eight analyzed patients in that study, the mtDNA deletion was present in satellite cells, but lost upon satellite cell activation and myoblast proliferation [16]. In contrast, MABs of a number of mtDNA point-mutation carriers in our study did display a high mtDNA mutation load (see Fig. 1). The low mtDNA mutation load in MABs of other point-mutation carriers are therefore unlikely a consequence of preferential expansion of MABs that contain a low mtDNA mutation load or due to selection against mutated MABs, although this cannot be fully excluded.

Satellite cell activation has been exploited to effectuate alterations in the mtDNA mutation load in patients [8, 13, 14, 33]. Based on our findings, mesoangioblasts could also contribute to mtDNA mutation load shifts upon muscle regeneration after muscle damage. Our data could also explain why this mtDNA mutation shift was not observed in all individuals. However, further research is warranted to assess the mtDNA mutation load in mesoangioblasts and satellite cells in parallel in vivo and ex vivo. Due to limitations in sample amount, this could unfortunately not be done in the present study. This information may also be of value to assess whether genetic drift, preferential replication of the mutated mtDNA in post-mitotic tissues, or other mechanisms underlie the mtDNA mutation load pattern in mesoangioblasts compared with the load in skeletal muscle. The low mutation load observed in MABs from half of the mtDNA point-mutation carriers, despite their high mutation load in skeletal muscle, is intriguing and might suggest selection either during embryonic development or later in life. Also, the distribution of the mtDNA mutation load in MABs is generally quite homogeneous, either low or high, but not uniformly distributed. A similar pattern was observed in myoblasts of m.8344A>G mutation carriers [26]. Possibly, MABs originate early during embryogenesis when the mtDNA copy number is very low, and solely wild-type or mutant mtDNA copies are transmitted. Further investigations, studying MABs during embryonic development, are warranted, but require identification of a specific MABs marker.

Mesoangioblasts are suitable as cell therapy medicinal product, and systemic intra-arterial delivery of allogeneic mesoangioblasts has been applied in a clinical study with DMD patients [25]. The natural appearance of mtDNA-mutation-free mesoangioblasts in more than half of the mtDNA mutation carriers offers the possibility of using mesoangioblasts for autologous stem cell therapy, which would circumvent adverse reactions to the transplant and exclude the use of immunosuppressive agents that are required for allogeneic transplantation. To this end, we assessed characteristics important for a cell therapy medicinal product, for MABs with low or absent mutation loads, derived from patients.

The proliferation capacity of these MABs is comparable to MABs derived from healthy individuals and DMD patients [22, 34]. Sampaolesi et al. demonstrated that a dog model of DMD clinically improved after systemic intra-arterial delivery of 5 × 10E7 MABs/kg [24]. For a 75-kg adult, ~ 3.8 × 10E9 MABs would be required. MAB isolation from a ~ 200-mg skeletal muscle biopsy would suffice to reach this target dose within 15 population doublings, thereby preserving proliferation capacity upon infusion. Immunophenotype analysis of the MABs verified that they were positive for CD13 and CD44 [22]. At early passage, such as p2, expression of (immature) endothelium markers CD31 and CD34, characteristic for pericytes, was observed, a trait lost during in vitro expansion [35]. Also, CD56+ satellite cells were also observed at early passages of some cultures. Since satellite cells cannot migrate from the bloodstream to the muscles, depletion of these cells is warranted for development of an intra-arterial delivery and can be successfully performed by magnetic-activated cell sorting using CD56 microbeads. MABs spontaneously differentiated into myotubes in vitro with varying efficiency (up to 37.1%). Similar to the results reported for MABs from healthy individuals and DMD patients [25, 34]. Statistical analysis of MAB myogenic capacity in relation to their mutation load and age at muscle biopsy demonstrated an interaction between the age and mutation load (Fig. 3), which confirms the negative impact aging has on the myogenic capacity of wild-type MABs [36]. All analyzed MABs displayed a normal mitochondrial OXPHOS capacity, at the exception of M2 and M11 MABs with a mtDNA mutation load of more than 80% that displayed deficient OXPHOS capacity. The mean mtDNA copy number per mesoangioblast is 148 ± 56, which is comparable to wild-type. Five cell lines displayed a mtDNA copy number higher than the mean ± 2 S.D., which cannot be directly related to their mutation or mutation load.

## Conclusion

Taken together, our data demonstrates that mtDNA-mutation-free MABs can be obtained from more than half of the patients. Their proliferation, myogenic differentiation capacity, and mitochondrial function are comparable with wild-type mesoangioblasts. This makes them good candidates for autologous transplantation to induce muscle regeneration, compatible with the shift of mtDNA mutation load due to eccentric/resistance exercise [8, 33]. As regular exercise is challenging for this patient group because of their disease status and lactate increase upon exercise, muscle regeneration by intra-arterial injection of mtDNA-mutation-free autologous mesoangioblasts could become a good alternative. In order to enable mesoangioblasts migration from the bloodstream to the muscles in this patient group, skeletal muscle inflammation and subsequent MABs migration can potentially be effectuated by a bout of eccentric exercise [37–39].

## Supplementary information

**Additional file 1: Table S1-S4** are available online

## Data Availability

All data generated and/or analyzed during this study are included in this manuscript and its supplementary information files.

## Abbreviations

MABs: Mesoangioblasts
mtDNA: Mitochondrial DNA

## References

[1] Gorman GS (2015). Prevalence of nuclear and mitochondrial DNA mutations related to adult mitochondrial disease. Ann Neurol.

[2] Taivassalo T (2003). The spectrum of exercise tolerance in mitochondrial myopathies: a study of 40 patients. Brain.

[3] Karppa M (2005). Spectrum of myopathic findings in 50 patients with the 3243A>G mutation in mitochondrial DNA. Brain.

[4] Gorman GS (2015). Perceived fatigue is highly prevalent and debilitating in patients with mitochondrial disease. Neuromuscul Disord.

[5] Majamaa K (1998). Epidemiology of A3243G, the mutation for mitochondrial encephalomyopathy, lactic acidosis, and strokelike episodes: prevalence of the mutation in an adult population. Am J Hum Genet.

[6] Petruzzella V (1994). Extremely high levels of mutant mtDNAs co-localize with cytochrome c oxidase-negative ragged-red fibers in patients harboring a point mutation at nt 3243. Hum Mol Genet.

[7] Nightingale H (2016). Emerging therapies for mitochondrial disorders. Brain.

[8] Murphy JL (2008). Resistance training in patients with single, large-scale deletions of mitochondrial DNA. Brain.

[9] Jeppesen TD (2006). Aerobic training is safe and improves exercise capacity in patients with mitochondrial myopathy. Brain.

[10] Walker DK (2012). PAX7+ satellite cells in young and older adults following resistance exercise. Muscle Nerve.

[11] Dreyer HC (2006). Satellite cell numbers in young and older men 24 hours after eccentric exercise. Muscle Nerve.

[12] Farup J (2015). Pericyte response to contraction mode-specific resistance exercise training in human skeletal muscle. J Appl Physiol.

[13] Fu K (1996). A novel heteroplasmic tRNAleu (CUN) mtDNA point mutation in a sporadic patient with mitochondrial encephalomyopathy segregates rapidly in skeletal muscle and suggests an approach to therapy. Hum Mol Genet.

[14] Shoubridge EA, Johns T, Karpati G (1997). Complete restoration of a wild-type mtDNA genotype in regenerating muscle fibres in a patient with a tRNA point mutation and mitochondrial encephalomyopathy. Hum Mol Genet.

[15] Clark KM (1997). Reversal of a mitochondrial DNA defect in human skeletal muscle. Nat Genet.

[16] Spendiff S (2013). Mitochondrial DNA deletions in muscle satellite cells: implications for therapies. Hum Mol Genet.

[17] Tedesco FS, Cossu G (2012). Stem cell therapies for muscle disorders. Curr Opin Neurol.

[18] Sancricca C (2010). Vessel-associated stem cells from skeletal muscle: from biology to future uses in cell therapy. World J Stem Cells.

[19] Roobrouck Valerie D., Clavel Carlos, Jacobs Sandra A., Ulloa-Montoya Fernando, Crippa Stefania, Sohni Abhishek, Roberts Scott J., Luyten Frank P., Van Gool Stefaan W., Sampaolesi Maurilio, Delforge Michel, Luttun Aernout, Verfaillie Catherine M. (2011). Differentiation Potential of Human Postnatal Mesenchymal Stem Cells, Mesoangioblasts, and Multipotent Adult Progenitor Cells Reflected in Their Transcriptome and Partially Influenced by the Culture Conditions. STEM CELLS.

[20] Sampaolesi M (2003). Cell therapy of alpha-sarcoglycan null dystrophic mice through intra-arterial delivery of mesoangioblasts. Science.

[21] Dellavalle A (2011). Pericytes resident in postnatal skeletal muscle differentiate into muscle fibres and generate satellite cells. Nat Commun.

[22] Dellavalle A (2007). Pericytes of human skeletal muscle are myogenic precursors distinct from satellite cells. Nat Cell Biol.

[23] Chen CW (2009). Perivascular multi-lineage progenitor cells in human organs: regenerative units, cytokine sources or both?. Cytokine Growth Factor Rev.

[24] Sampaolesi M (2006). Mesoangioblast stem cells ameliorate muscle function in dystrophic dogs. Nature.

[25] Cossu G (2015). Intra-arterial transplantation of HLA-matched donor mesoangioblasts in Duchenne muscular dystrophy. EMBO Mol Med.

[26] Boulet L, Karpati G, Shoubridge EA (1992). Distribution and threshold expression of the tRNA (Lys) mutation in skeletal muscle of patients with myoclonic epilepsy and ragged-red fibers (MERRF). Am J Hum Genet.

[27] Tonlorenzi, R., et al., Isolation and characterization of mesoangioblasts from mouse, dog, and human tissues*.* Curr Protocols Stem Cell Biol, 2007. Chapter 2: p. Unit 2B 1. 10.1002/9780470151808.sc02b01s3 18785178

[28] Quattrocelli M (2012). Mouse and human mesoangioblasts: isolation and characterization from adult skeletal muscles. Methods Mol Biol.

[29] Sallevelt SC (2013). Preimplantation genetic diagnosis in mitochondrial DNA disorders: challenge and success. J Med Genet.

[30] Akaike H, Petrov BN, Csàki F (1973). Information theory and an extension of the maximum likelihood principle. Second International Symposium on Inference Theory.

[31] Ihaka R, Gentleman R (1996). R: a language for data analysis and graphics. J Comput Graphics Stat.

[32] Lindsey J, 2 (1999). Models for repeated measurements.

[33] Taivassalo T (1999). Gene shifting: a novel therapy for mitochondrial myopathy. Hum Mol Genet.

[34] Meng J (2011). Contribution of human muscle-derived cells to skeletal muscle regeneration in dystrophic host mice. PLoS One.

[35] Morosetti R (2011). Mesoangioblasts of inclusion-body myositis: a twofold tool to study pathogenic mechanisms and enhance defective muscle regeneration. Acta Myologica.

[36] Rotini Alessio, Martínez-Sarrà Ester, Duelen Robin, Costamagna Domiziana, Di Filippo Ester Sara, Giacomazzi Giorgia, Grosemans Hanne, Fulle Stefania, Sampaolesi Maurilio (2018). Aging affects the in vivo regenerative potential of human mesoangioblasts. Aging Cell.

[37] Valero MC (2012). Eccentric exercise facilitates mesenchymal stem cell appearance in skeletal muscle. PLoS One.

[38] Galvez BG (2006). Complete repair of dystrophic skeletal muscle by mesoangioblasts with enhanced migration ability. J Cell Biol.

[39] Nederveen JP (2018). The influence of capillarization on satellite cell pool expansion and activation following exercise-induced muscle damage in healthy young men. J Physiol.

