# Supplementary material for: Healthy, mtDNA-mutation free mesoangioblasts from mtDNA patients qualify for autologous therapy
Source: Stem Cell Res Ther. 2019 Dec 21;10:405. doi: 10.1186/s13287-019-1510-8 (PMC6925445; doi:10.1186/s13287-019-1510-8)
Supplement: Supplementary file 1 — Additional file 1: Table S1-S4 are available online [file 13287_2019_1510_MOESM1_ESM.pdf]

Table S1. Primers, PCR conditions, restriction digestion enzyme and expected sizes for all analyzed mtDNA pointmutations and mtDNA copy number analysis

Mutation	Sequence 5'-3'	Primer	Modification primer	Analysis method	Annealing temperature	MgCl2 (mM)	Size amplicon (bp)	Restriction enzyme	Size mutant fragment	Size wildtype fragment
	CAACTTAGTATTATACCCACAC	For								
m.3243A>G	CAACTTAGTATTATACCCACAC	For	Fam label	Genescan	53°C	2,00	195	*Hae* III	48	122
	TTTCGTTCGGTAAGCATTAG	Rev								
	AGGACAAGAGAAATAAGGCC	For								
m.3271T>C	AGGACAAGAGAAATAAGGCC	For	Fam label	Genescan	53°C	2,00	171	*Afl* II	140	167
	TAAGAAGAGGAATTGAACCTCTGACCTTAA	Rev								
	TACTTCACAAAGCGCCTTCC	For								
m.3291T>C	TACTTCACAAAGCGCCTTCC	For	Fam label	Genescan	60°C	1,50	399	*Tsp509* I	233	136
	ATGGTGAGAGCTAAGGTCGG	Rev								
	TGTAAAACGACGGCCAGTAACCAACACCTCTTTA	For								
m.8363G>A	TGTAAAACGACGGCCAGT	For	Fam label	Genescan	60°C	1,50	142	*HpH* I	92	38
	TTTGGTGAGGGAGGTAGGTG	Rev								
	TACGAACGCACTCACAGTC**G**	For wt								
m.11778G>A	TACGAACGCACTCACAGTC**A**	For mut		quantitative PCR	67°C		142			
	CAGAGAGTTCTCCCAGTAGGTTAAT	Rev								
D-loop	CATCTGGTTCCTACTTCAGGG	For		quantitative PCR	60°C		104			
	TGAGTGGTTAATAGGGTGATAGA	Rev								
B2M	TGCTGTCTCCATGTTTGATGTATCT	For		quantitative PCR	60°C		85			
	TCTCTGCTCCCCACCTCTAAGT	Rev								
mtDNA large-scale deletions	CCGCACAAGAGTGCTACTCTCCTC	For		0,7% agarose gel analysis	72°C		16569			
	GATATTGATTTCACGGAGGATGGTG	Rev								
m8482_m.13460 4977bp deletion	CCCTCTAGAGCCCACTG	For		1,0% agarose gel analysis	60°C		475			
	AAATCCTGCGAATAGGCTTCC	Rev								

S2. Validation quantitative analysis of m.11778G>A mutation load using qPCR

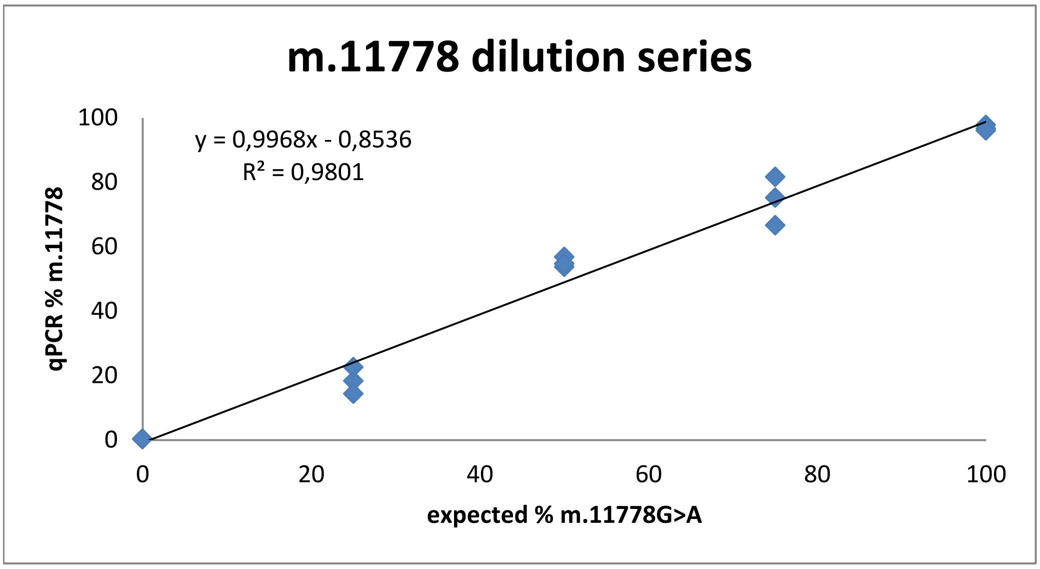

Table S3. immunophenotype analysis

	**CD13 (>90%)**	**CD44 (>90%)**	**CD31 (<5%)**	**CD34 (<5%)**	**CD45 (<5%)**	**CD56 (<5%)**
M137 p6	100,0	99,8	0,6	3,1	1,2	4,3
M02 p6	99,8	99,7	0,2	0,1	0,2	43,8
M06 p12	100,0	100,0	1,3	0,2	0,1	30,0
M07 p5	99,9	99,6	0,1	0,1	0,2	31,5
M08 p6	98,4	98,2	1,3	0,8	1,2	8,7
M11 p9	100,0	100,0	1,0	0,7	0,7	56,3
M16 p	97.7	98.8	5.9	3.4	4.3	2,1
M18 p12	99,9	99,7	0,1	0,1	0,1	0,4
M28 p7	99,9	99,9	0,6	0,1	0,1	0,1
M32 p7	99,8	99,9	0,2	0,1	0,1	0,7
M33 p7	99,0	99,2	0,8	0,1	0,1	4,4
M34 p2	99,5	97,3	0,9	6,3	1,5	15
M34 p8	99,6	99,6	0,5	0,1	0,2	0,3
mean	99,65	99,41	0,63	0,98	0,48	15,20
stdev	0,49	0,83	0,44	1,88	0,53	18,97

Table S4. In vitro myogenic capacity

Patient ID	Age	Gender	mtDNA mutation	mtDNA mutation load in sk.muscle (%)	mtDNA mutation load in MABs (%)	mean myogenic differentiation capacity (%)
M10	33	F	3243	28	1	16,9
M137	58	M	3243	80	22	14,1
M32	40	M	3243	80	67	4,1
M5	27	F	3243	40	9	13,5
M6	21	M	3243	93	3	37,1
M9	59	F	3243	15	2	21
M2	22	F	3271	100	96	8,7
M22	51	F	3271	73	41	3,3
M11	11	F	3291	94	73	3,7
M1	37	M	8363	46	1	30,8
M18	35	F	8363	87	78	12,9
M8	40	F	8363	51	6	32,0
M34	63	M	11778	89	68	4,6
M134	60	F	del	60	1	4,6
M24	21	F	del	ND	1	11,1
M28	58	M	del	ND	1	0,0
M33	66	F	del	45-50	0	12,9
M7	42	M	del	60-70	0	26,4

ND: not determined
